# Laubry–Pezzi Syndrome: A Case Report of a Rare Entity

**DOI:** 10.7759/cureus.82592

**Published:** 2025-04-19

**Authors:** Belen Domingo Cruz Hernandez, José Ricardo Chávez Delgado, Karina Lizbeth Lara Sampayo, José Luis Ortiz Fernández, Mauricio Alejandro Lozano Rodríguez, Paulina Gutiérrez Valladares, Brittani Trejo, Daniel Herrera Hernández

**Affiliations:** 1 Medicine, Universidad Xochicalco, Ensenada, MEX; 2 Cardiology, UMAE Hospital de Cardiología No. 34, Instituto Mexicano del Seguro Social, Monterrey, MEX; 3 Medicine, Hospital General de Zona con Medicina Familiar No. 2, Instituto Mexicano del Seguro Social, Monterrey, MEX; 4 Medicine and Surgery, Hospital General de Gómez Palacio, Durango, MEX; 5 Medicine and Surgery, Universidad Autónoma de Aguascalientes, Aguascalientes, MEX; 6 Medicine, Universidad de Guadalajara, Guadalajara, MEX; 7 Medicine and Surgery, Universidad Latina de Mexico, Celaya, MEX; 8 Medicine and Surgery, Hospital General Regional No. 1, Instituto Mexicano del Seguro Social, Tijuana, MEX

**Keywords:** aortic regurgitation, cardiothoracic and vascular surgery research, laubry-pezzi syndrome, surgical case reports, ventricular septal defect

## Abstract

Laubry-Pezzi syndrome is a rare congenital cardiac condition characterized by a ventricular septal defect (VSD) and aortic regurgitation (AR) due to aortic cusp prolapse. Although the management of this syndrome is not well-established, early closure of the VSD is recommended to prevent or minimize the progression of AR. This report presents a case of a 23-year-old female diagnosed with Laubry-Pezzi syndrome. Our findings emphasize the importance of early diagnosis and prompt intervention to reduce the risk of complications, such as infective endocarditis and progressive aortic valve dysfunction. This report underscores the need for tailored management strategies, with the possibility of aortic valve repair or replacement depending on the severity of AR and VSD.

## Introduction

Laubry-Pezzi syndrome is a rare congenital cardiac anomaly characterized by a ventricular septal defect (VSD) associated with aortic regurgitation (AR), typically resulting from prolapse of an aortic cusp into the VSD. First described in 1921 by Charles Laubry and Cesare Pezzi, the syndrome represents a progressive pathological process, often developing during adolescence or early adulthood [1,2]. The condition is most frequently associated with perimembranous or subarterial VSDs, which create a Venturi effect that draws the aortic cusp into the defect, eventually leading to valvular incompetence [3,4].

Although it is considered a rare disease, the incidence of aortic cusp prolapse among patients with perimembranous VSD has been reported to be between 5% and 8% [3,5]. The right coronary cusp is most commonly affected, followed by the noncoronary cusp. Diagnosis relies heavily on echocardiography, which allows detailed assessment of the septal defect, valve morphology, and degree of AR [4].

Due to the progressive nature of the lesion, early surgical intervention is often required. Failure to close the VSD promptly can lead to irreversible valve damage, necessitating valve repair or replacement [1,5]. While isolated VSD closure may be sufficient in some cases, advanced disease frequently requires aortic valve intervention. This case report aims to illustrate the clinical progression and surgical management of a young adult with Laubry-Pezzi syndrome and provide a comprehensive review of current literature on the topic.

## Case presentation

A 23-year-old female with a known history of VSD presented to our cardiology clinic with progressive dyspnea over the past year. She was born full-term via spontaneous vaginal delivery, with no perinatal complications reported at birth. However, she experienced psychomotor developmental delay attributed to neonatal hypoxia. Her medical history was notable for the diagnosis of a perimembranous VSD at age 11, which had been monitored without surgical intervention.

The patient denied a history of chest pain, syncope, palpitations, fever, or previous hospitalizations. Family history was relevant for type 2 diabetes in her father and hypertension in her maternal grandmother. She had no history of smoking, alcohol use, surgeries, trauma, transfusions, or known allergies.

On physical examination, her vital signs were stable. Cardiac auscultation revealed a grade III/VI holosystolic murmur best heard at the left lower sternal border and a diastolic murmur at the right upper sternal border. There were no signs of peripheral edema, cyanosis, or jugular venous distension.

**Figure 1:**
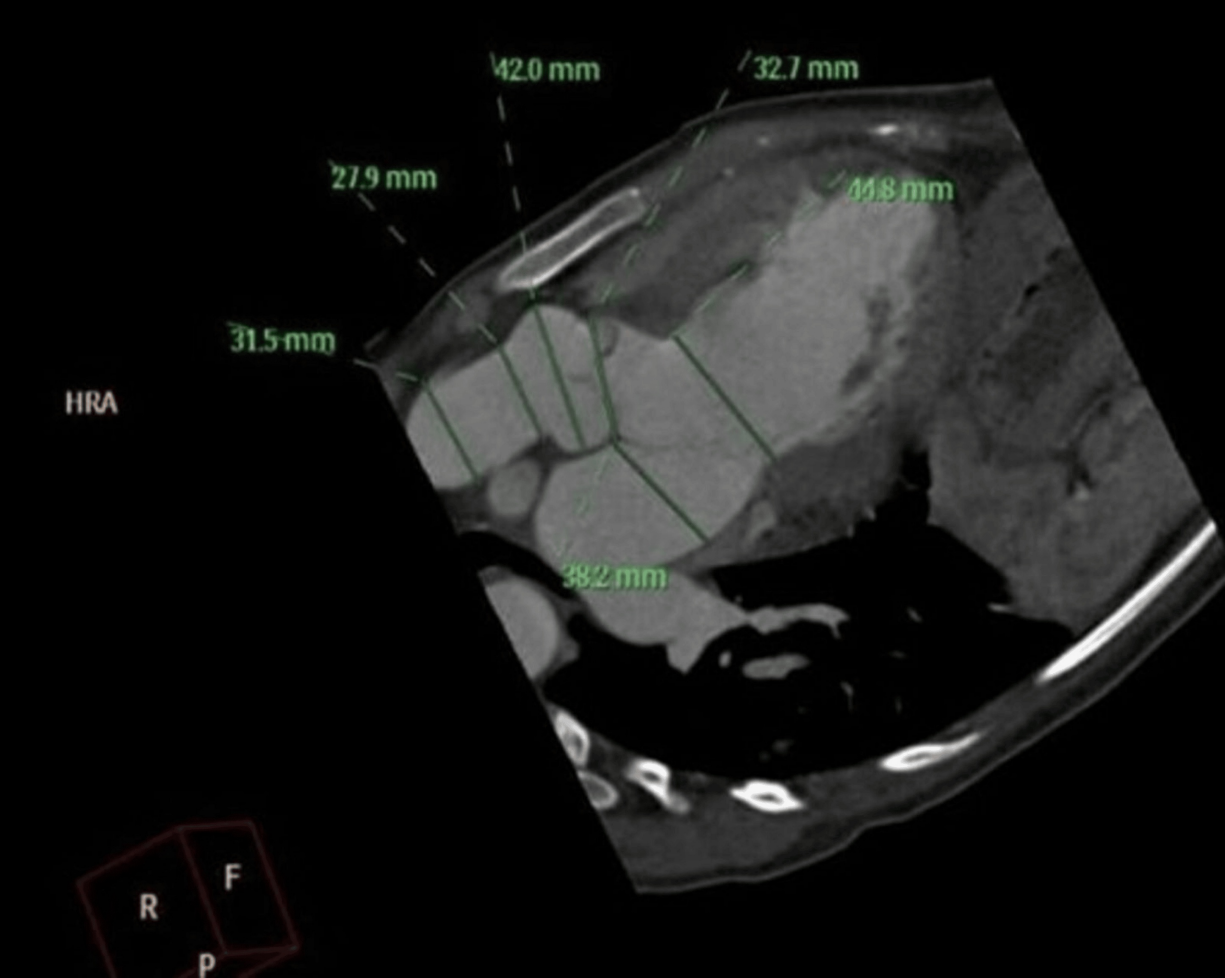
Perimembranous ventricular septal defect, dilatation of the aortic root and descending aorta, pseudocoarctation of the aorta, and severe LV dilatation (left ventricular ejection fraction: 51%).

An initial transthoracic echocardiogram (TTE) performed in March 2023 demonstrated a left ventricular (LV) dilation with preserved ejection fraction, a perimembranous VSD, and signs of aortic cusp prolapse causing moderate-to-severe AR. Angio-CT in June 2023 revealed dilatation of the aortic root and descending aorta, pseudocoarctation of the aorta, and LV enlargement (left ventricular ejection fraction [LVEF]: 51%) (Figure 1). A follow-up TTE in July 2023 showed worsening AR, a ruptured right coronary sinus with a 7-mm communication into the right ventricle, moderate pericardial effusion without tamponade, and improved LVEF at 61%.

**Figure 2:**
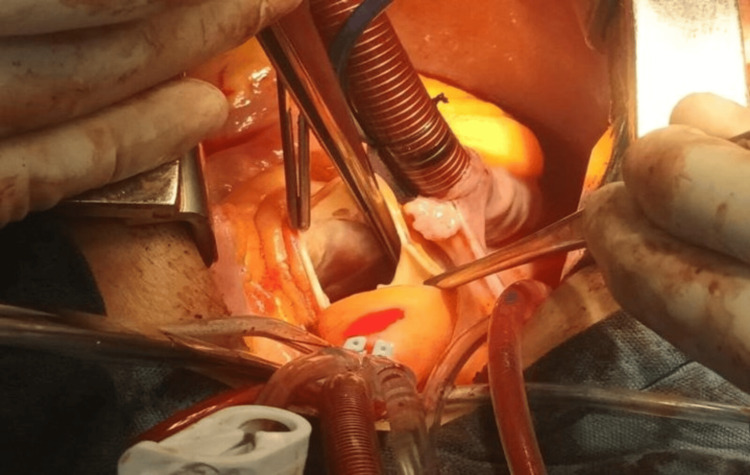
Sternum of regular quality, trileaflet aortic valve, non-coapt dysplastic valves, right valve with calcium plaque extending to commissure with left valve, 12-mm perimembranous ventricular septal defect, and closed pleura.

Given the clinical and imaging findings, the patient was referred for surgical evaluation. In a multidisciplinary cardiac surgery meeting, the decision was made to proceed with aortic valve replacement and repair of the sinus of Valsalva. Intraoperatively, the sternum appeared normal. The aortic valve was tricuspid, dysplastic, and non-coapting, with a calcium plaque on the right cusp extending to the commissure. A 12-mm perimembranous VSD was confirmed and repaired (Figure 2).

Postoperatively, the patient had an uneventful recovery. Follow-up echocardiography demonstrated resolution of AR and normalization of LV dimensions. She remained asymptomatic at the three-month outpatient follow-up, with no evidence of pericardial effusion or conduction abnormalities. Histopathological examination confirmed degenerative changes in the aortic valve consistent with chronic prolapse and regurgitation.

## Discussion

Laubry-Pezzi syndrome is an uncommon but clinically significant congenital cardiac condition, in which a VSD is complicated by prolapse of an aortic valve cusp, resulting in progressive AR. This syndrome typically develops in patients with perimembranous or subarterial VSDs, where the Venturi effect pulls the cusp into the defect [3].

The most frequently affected cusp is the right coronary cusp, although the noncoronary cusp may also be involved [1]. This leads to valvular incompetence, with resultant LV volume overload, progressive dilation, and eventual systolic dysfunction if left untreated. While many patients are asymptomatic during early stages, progressive exertional dyspnea, palpitations, and heart murmurs may develop as AR worsens [1,3].

Diagnosis is primarily made with TTE, which can detect the VSD, cusp prolapse, and severity of regurgitation [4]. Advanced imaging such as transesophageal echocardiography (TEE) and cardiac CT can provide further anatomical detail. In our case, both TTE and angio-CT were critical for delineating the extent of the defect and planning surgical intervention.

There is no consensus on the optimal timing of surgical intervention in Laubry-Pezzi syndrome. However, early closure of the VSD - prior to the onset of significant AR - is generally recommended [2,4]. Once AR develops, isolated closure of the VSD may not be sufficient, and aortic valve repair or replacement becomes necessary. Surgical options depend on the degree of cusp involvement and patient age, with valve-sparing techniques preferred in younger individuals to avoid long-term anticoagulation [5].

In this case, the patient required both VSD closure and valve replacement due to advanced valvular degeneration. The use of multidisciplinary decision-making and individualized planning contributed to a favorable outcome.

Comparative literature reveals variable approaches and outcomes. Zniber et al. reported a case of Laubry-Pezzi syndrome in an eight-year-old boy who underwent a Ross procedure and VSD repair with long-term recovery [6]. Boukhmis and Nouar described the surgical treatment of Laubry-Pezzi syndrome complicated by persistent left superior vena cava and airlock during bypass, emphasizing the importance of careful intraoperative planning [7]. Similarly, their 2022 follow-up publication reiterated the challenges posed by extracardiac venous anomalies in similar contexts [8].

Sbizzera et al. reported long-term complications including aortic root pseudoaneurysm and residual VSD following childhood correction of Laubry-Pezzi syndrome, reinforcing the need for lifelong surveillance [9]. Finally, Pontailler et al. demonstrated the feasibility and effectiveness of aortic valve-sparing techniques, highlighting anatomical restoration through a transaortic approach in pediatric patients [10].

These reports underscore the heterogeneity in clinical presentation, surgical options, and long-term outcomes associated with Laubry-Pezzi syndrome, justifying a tailored approach and long-term follow-up.

## Conclusions

Laubry-Pezzi syndrome represents a rare but potentially progressive complication of congenital VSDs. The development of aortic valve prolapse and regurgitation significantly increases the risk of heart failure, requiring early recognition and timely intervention. TTE plays a central role in diagnosis and follow-up, allowing for the evaluation of both the septal defect and valve morphology. Surgical management must be individualized based on anatomical findings, degree of valvular damage, and patient-specific factors. This case emphasizes the importance of echocardiographic surveillance and a multidisciplinary approach to optimize surgical outcomes and long-term prognosis in patients with this complex condition.
